# Pure-high-even-order dispersion bound solitons complexes in ultra-fast fiber lasers

**DOI:** 10.1038/s41377-024-01451-z

**Published:** 2024-05-06

**Authors:** Ying Han, Bo Gao, Honglin Wen, Chunyang Ma, Jiayu Huo, Yingying Li, Luyao Zhou, Qi Li, Ge Wu, Lie Liu

**Affiliations:** 1 College of Communication Engineering, Jilin University, Changchun, 130012 China; 2 Research Center of Circuits and Systems, Peng Cheng Laboratory, Shenzhen, 518055 China; 3 International Collaborative Laboratory of 2D Materials for Optoelectronics Science and Technology of Ministry of Education, Institute of Microscale Optoelectronics, Shenzhen University, Shenzhen, 518060 China; 4 College of Electronic Science and Engineering, Jilin University, Changchun, 130012 China

**Keywords:** Ultrafast lasers, Fibre lasers

## Abstract

Temporal solitons have been the focus of much research due to their fascinating physical properties. These solitons can form bound states, which are fundamentally crucial modes in fiber laser and present striking analogies with their matter molecules counterparts, which means they have potential applications in large-capacity transmission and all-optical information storage. Although traditionally, second-order dispersion has been the dominant dispersion for conventional solitons, recent experimental and theoretical research has shown that pure-high-even-order dispersion (PHEOD) solitons with energy-width scaling can arise from the interaction of arbitrary negative-even-order dispersion and Kerr nonlinearity. Despite these advancements, research on the bound states of PHEOD solitons is currently non-existent. In this study, we obtained PHEOD bound solitons in a fiber laser using an intra-cavity spectral pulse shaper for high-order dispersion management. Specifically, we experimentally demonstrate the existence of PHEOD solitons and PHEOD bound solitons with pure-quartic, -sextic, -octic, and -decic dispersion. Numerical simulations corroborate these experimental observations. Furthermore, vibrating phase PHEOD bound soliton pairs, sliding phase PHEOD bound soliton pairs, and hybrid phase PHEOD bound tri-soliton are discovered and characterized. These results broaden the fundamental understanding of solitons and show the universality of multi-soliton patterns.

## Abstract (web summary)

The pure-high-even-order dispersion bound solitons complexes, which build up in high-order dispersion management ultra-fast fiber laser, are revealed both theoretically and experimentally, enriching the framework towards multi-soliton complexes.

## Graphical abstract

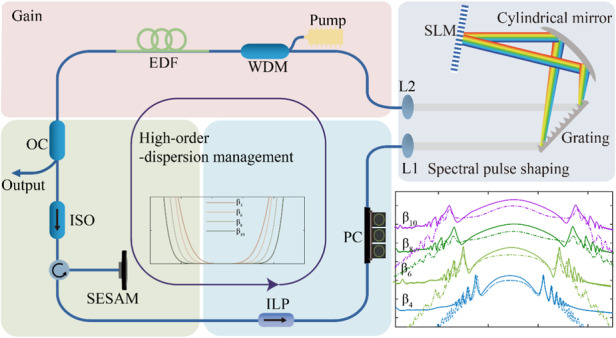

## Introduction

The interplay between dispersion and nonlinearity plays a crucial role in the dynamics in passively mode-locked fiber lasers, and the research on dispersion management has a long history^1^. Considering that conventional solitons arise from the balance between negative second-order dispersion (*β*_*2*_) and self-phase modulation (SPM), the management of *β*_*2*_ has been the primary focus of previous research^2^. Various solitons can be achieved through *β*_*2*_ management using dispersion-compensated fibers, chirped mirrors, G-T mirrors, chirped fiber Bragg gratings, prism pairs, or grating^3^. Historically, high-order dispersion was considered detrimental, leading to soliton instabilities or energy loss^4–6^. However, this perspective shifted in 2016 when Redondo et al. discovered “pure-quartic solitons (PQSs)” in photonic crystal waveguides, which emerged from the balance of SPM and negative fourth-order dispersion (*β*_*4*_)^7^. The pulse energy of PQSs is proportional to the third power of inverse pulse duration (the energy-width scaling), implying that the energy of PQSs can be significantly higher than conventional solitons for the same pulse duration. For the fiber laser systems, research on PQSs began in 2018, Redondo et al. designed a micro-structured fiber to obtain PQSs, laying the groundwork for PQS fiber laser research^8^. The nonlinear Schrödinger equation (NLSE) and the cubic-quintic Ginzburg-landau equation with *β*_*2*_ and *β*_*4*_ have the analytical solution, and such combinations can enhance the performance of fiber lasers^9,10^. NLSE and its extended form are fundamental equations for describing pulse transmission in fibers and are ideal for modeling fiber lasers^11^. These numerical results provide theoretical support for constructing PQS fiber lasers. Redondo et al. constructed the PQSs passively mode-locked fiber laser in 2020 and analyzed the oscillation characteristics of PQSs through simulation^12,13^. In subsequent research, they confirmed that conventional solitons and PQSs are just two lowest-order members of an infinite hierarchy of solitons arising from the interaction of nonlinearity and negative pure-high-even-order dispersion (PHEOD). Controlling high-order dispersion provides a new method to access an infinite family of nonlinear pulses^14^. In contrast, the effect of odd-order-dispersion (e.g. third-order dispersion^15^) differs significantly from that of even-order-dispersion, as the group velocity dependence associated with odd-order-dispersion is not monotonic^16^, making it challenging to form pure-odd-order-dispersion solitons. Moreover, considering the energy-width scaling of PHEOD solitons^14^, it is necessary to investigate the impact of high-even-order dispersion on PHEOD solitons.

The existence and stability of bound states, comprising two or more solitons, are dictated by the separations and phase differences among the constituent solitons^17^. Passively mode-locked fiber lasers offer a highly efficient platform for bound solitons generation. Numerous experiments conducted in such systems have yielded stationary bound solitons with fixed phase differences^17^. However, the variation of the phase difference enables the observation of complex bound soliton dynamics^18^, such as the vibrating phase, stepwise phase, and sliding phase^19–21^. In recent years, the investigation of these transient dynamics has been facilitated by the application of the dispersive Fourier transform (DFT) technique^22,23^. Various bound solitons introduced above were predominantly classified based on their evolution traces of phase difference and separation. On this basis, energy evolution is introduced to establish the relationship between phase evolution and energy change of solitons^24^. In particular, the ability to produce on-demand bound solitons is crucial for optical data-processing schemes^25^, optical switching^26^, storage^27^, and soliton trapping^28^. For example, the generation of four distinct types of bound solitons, each with different phase differences, can be regulated by manipulating the energy exchange between the solitons^29^. Researchers have divided the dispersion into a real part and an imaginary part to control the separation by encoding the phase and amplitude in the hologram of the liquid crystal spatial light modulator^30^, thereby realizing quaternary coding by the above-mentioned bound solitons^29,30^.

These extensive explorations have demonstrated the universality of bound soliton dynamics in passively mode-locked fiber lasers. The intriguing questions that arise are whether PHEOD bound solitons can exist and what properties they would have. The unique oscillatory tails of PQSs could lead to novel dynamics in terms of relative motion and energy exchange^12,31–34^. We have demonstrated the effect of gain on creeping bound PQSs in NLSE-based fiber laser cavities^35^, providing a new perspective for the study of bound PQSs. Subsequently, Song et al. confirmed that bound PQSs exhibit periodic pulsating similar to that of pulsating conventional bound solitons with the increase of *β*_*4*_^36^. However, there is currently no experimental research on PHEOD bound soliton fiber laser, making it desirable to discover the nonlinear dynamics of PHEOD solitons and their bound states.

In this paper, we incorporate a pulse shaping structure into a semiconductor saturable absorber mirror (SESAM) passively mode-locked fiber ring cavity to realize the compensation of *β*_*2*_ and third-order dispersion (*β*_*3*_). On this basis, we introduced large negative fourth-order (*β*_*4*_), sixth-order (*β*_*6*_), eighth-order (*β*_*8*_), and tenth-order (*β*_*10*_) dispersion to achieve pure-quartic, -sextic, -octic, and -decic solitons, respectively. By adjusting the intra-cavity polarization controller, we can generate pure-octic bound solitons with varying soliton numbers. These results were subsequently verified through simulation, and the characteristics of sliding phase PHEOD bound soliton pairs, vibrating phase PHEOD bound soliton pairs, and hybrid phase PHEOD bound tri-solitons were analyzed, proving that PHEOD bound solitons exhibit similar dynamics to conventional bound solitons. All the results provide new insights into the dynamics of PHEOD bound solitons and enrich the framework towards multi-soliton complexes.

## Results

### Experimental results

**Fig. 1:**
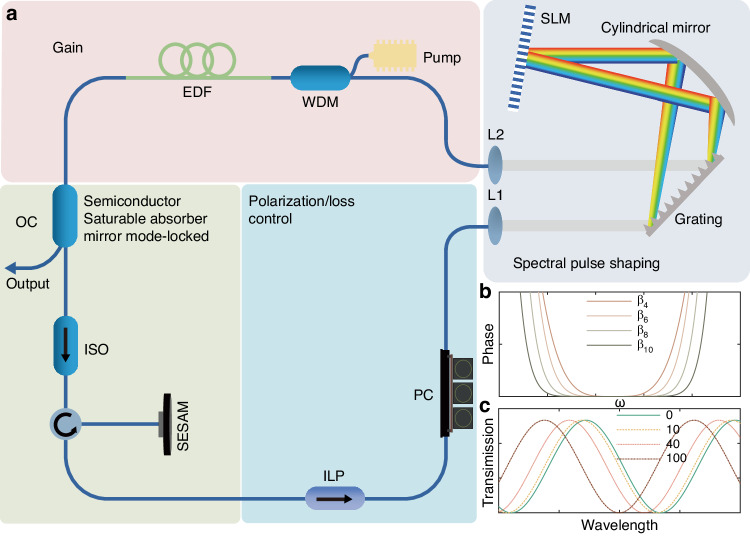
**a** Schematic diagram of the PHEOD soliton passively mode-locked fiber laser. **b** Phase profile induced by spectral pulse shaping. **c** Transmission of nonlinear polarization rotation

The configuration of the PHEOD soliton passively mode-locked fiber laser is depicted in Fig. 1a. It comprises four components: gain, saturable absorber, polarization/loss control, and spectral pulse shaping. A commercial SESAM is used to achieve passive mode-locking, and a three-ring polarization controller (PC) is employed to adjust intra-cavity loss (Fig. 1c). The pigtail of the wavelength division multiplexer is HI1060, while the pigtails of other intra-cavity devices are SMF28e. The total fiber length of the fiber ring cavity is 26.6 m, corresponding to a repetition rate of 7.9475 MHz. Spectral pulse shaping can be straightforwardly implemented in a fiber laser cavity. By implementing the phase profile depicted in Fig. 1b, the inherent *β*_*2*_ and *β*_*3*_ of the fiber cavity can be compensated, and the management of large negative high-even-order dispersion can be achieved. Further details about the fiber cavity and the measurement systems are described in the “Materials and methods” section.

### Pure-high-even-order dispersion single solitons

When propagation within the cavity, solitons experience periodic perturbations and undergo reshaping to preserve their shape. During the reshaping process, solitons emit energy via dispersive wave radiation, generating analogous linear dispersion waves with each round-trip (RT). However, phase interference only transpires at specific frequencies, leading to the resonance enhancement of dispersion waves. This results in the formation of peaks in the soliton spectra, known as Kelly sidebands. The positions of these peaks offer insights into intra-cavity dispersion. Therefore, we can estimate the dispersion of a fiber cavity by analyzing the sideband positions in the soliton spectra. Constructive interference occurs as ${\beta }_{soliton}-{\beta }_{dispersive}=2\pi m/L$ (*m* is a positive integer). kth linear dispersion waves satisfied the condition of${\beta }_{dispersive}=-|{\beta }_{k}|{(\omega -{\omega }_{0})}^{k}/k!$, while pure-high-even-order dispersion solitons have constant dispersion ${\beta }_{soliton}={C}_{k}|{\beta }_{k}|/{\tau }^{k}$ over the entire bandwidth. *C*_*k*_ represents the unit constant related to the dispersion order^9^. Thus, the position of the *m*-th spectrum sideband can be expressed as:

1 $${\omega }_{m}=\pm \frac{1}{\tau} {\left[k!\left(\frac{2m\pi {\tau }^{k}}{|{\beta }_{k}|L}-{C}_{k}\right)\right]}^{1/k}$$

**Fig. 2:**
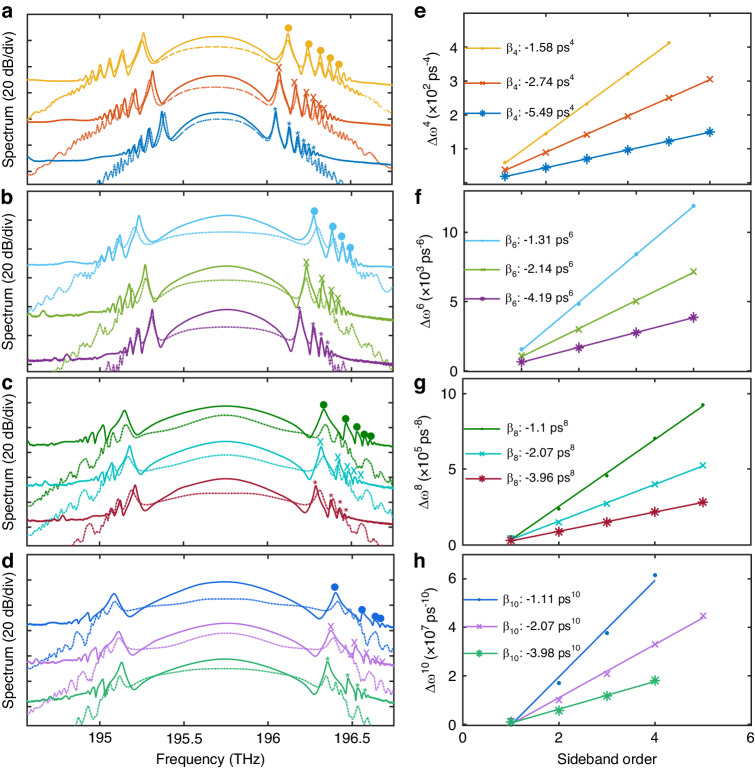
Measured (solid lines) and simulated (dashed lines) PHEOD solitons with different dispersion. **a** Pure-quartic soliton spectra when taking *β*_*4*_ as -1.596 ps^4^ (yellow), –2.66 ps^4^ (orange), –5.32 ps^4^ (dark blue). **b** Pure-sextic soliton spectra when taking *β*_*6*_ as –1.33 ps^6^ (light blue), –2.128 ps^6^ (light green), –4.256 ps^6^ (purple). **c** Pure-octic soliton spectra when taking *β*_*8*_ as –1.064 ps^8^ (dark green), –2.128 ps^8^ (magenta blue), –3.99 ps^8^ (red). **d** Pure-decic soliton spectra when taking *β*_*10*_ as –1.064 ps^10^ (dark blue), –2.128 ps^10^ (light purple), –3.99 ps^10^ (light green). The colored circles, cross, and asterisks show the kth power of the sideband positions versus sideband order of the (**e**) pure-quartic, (**f**) pure-sextic, (**g**) pure-octic, and (**h**) pure-decic soliton spectra, solid lines correspond to a linear fit, the legend provides the calculated *β*_*k*_

Equation (1) demonstrates that the *k*-power associated with two adjacent sidebands within the pure-high-k-order soliton spectrum is constant$2\pi {\rm{k}}!/(|{\beta }_{k}|L)$, regardless of the value of *C*_*k*_^9^. To corroborate this prediction, we executed a series of measurements on soliton spectra, systematically varying the dispersion coefficients *β*_*4*_, *β*_*6*_, *β*_*8*_, and *β*_*10*_. Subsequently, we scrutinized the positions of high-frequency sidebands within these spectra. The corresponding experimental (solid lines) and simulation (dashed lines) results are present in Fig. 2a–d. Figure 2a portrays the spectra of pure-quartic soliton under three different *β*_*4*_ values. Figure 2b–d delineate the corresponding spectra of pure-sextic, pure-octic, and pure-decic solitons under varying *β*_*6*_, *β*_*8*_, and *β*_*10*_ values, respectively. The circles, crosses, and asterisks denote the positions of high-frequency sidebands at different dispersion coefficients. Figure 2e–h depict the kth power of the measured sideband position as a function of sideband order for the corresponding PHEOD soliton spectra in Fig. 2a–d. The sideband spacing follows the expected linear relationship in all cases. It is important to note that the agreement between the calculated values according to Eq. (1) based on experiment results, the simulation results, and the high-order dispersion values applied to the spectral pulse shaping structure (further details are described in the “Materials and methods” section) is so high that it is difficult to distinguish them. Therefore, the corresponding simulation results are not present in Fig. 2e–h, but give the corresponding calculated values from experiment results. Results for low-frequency sidebands (not shown in the figure) also closely align with theoretical values. Due to the use of a high-power data set amplifying the noise, the agreement between the measured results and the expected results is remarkable, thus confirming the type and magnitude of intra-cavity dispersion^9,13,16^.

### Pure-high-even-order dispersion bound multi-solitons

**Fig. 3:**
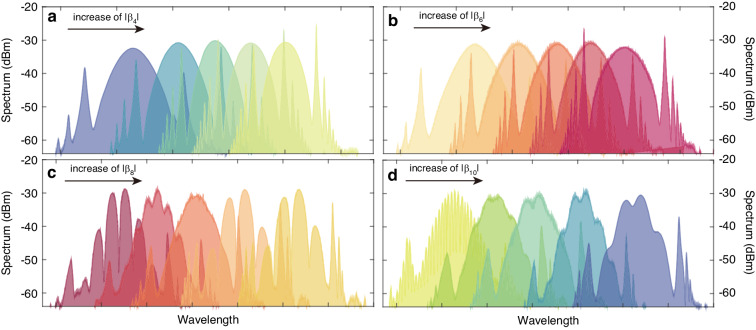
Measured spectra with fixed pump power and PC blade direction and taken intra-cavity net high-even-order dispersion (from left to right) when taking (**a**) *β*_*4*_ as –0.266 ps^4^, –0.78 ps^4^, –2.086 ps^4^, –2.86 ps^4^, –3.64 ps^4^; (**b**) *β*_*6*_ as –0.266 ps^6^, –1.04 ps^6^, –2.08 ps^6^, –2.86 ps^6^, –3.38 ps^6^; (**c**) *β*_*8*_ as –0.266 ps^8^, –0.532 ps^8^, –1.04 ps^8^, –2.08 ps^8^, –3.64 ps^8^; (**d**) *β*_*10*_ as –0.266 ps^10^, –1.56 ps^10^, –2.34 ps^10^, –3.12 ps^10^, –3.64 ps^10^

In the presence of high pump power, solitons will split due to the peak power clamping effect, transitioning the laser from a single soliton state to a multi-soliton state. Bound solitons, a captivating aspect of soliton dynamics, are energy-quantized and bounded together due to the balance of repulsive and attractive forces between solitons^37^. Researchers globally have extensively studied bound solitons and reported their generation in normal dispersion, near-zero dispersion, and anomalous dispersion fiber lasers^17^. However, there are no experimental reports about PHEOD bound solitons in passively mode-locked fiber lasers to date. In our study, we experimentally obtained modulated soliton spectra (bound solitons) by high-order dispersion management through the spectral pulse shaping structure, while keeping the pump power and the blade direction of the PC unchanged. Corresponding experimental results are presented in Fig. 3. Figure 3a, b indicates that the changes of intra-cavity |*β*_*4*_| and |*β*_*6*_| are not easy to form modulated spectra (bound solitons). Soliton spectra in Fig. 3d exhibit modulation characteristics as the increase of intra-cavity |*β*_*10*_ | , but the change is not significant. However, in Fig. 3c, the increase of |*β*_*8*_| causes a significant variation in soliton spectra (from non-modulation to modulation). That is, compared to other-order-dispersions, *β*_*8*_ is more likely to cause alteration in soliton spectra and is more conducive to exploring bound solitons. This may be due to the formation of bound solitons related to the interaction between solitons^38^, the time-domain tails^33,39–42^, and the spectral sidebands^43,44^. PHEOD soliton spectra have a series of sidebands and the time-domain tailing induced by high-even-order dispersion makes PHEOD soliton easier to have short-range interactions to form bound states. Our analysis is focused on the impact of intra-cavity net *β*_*8*_ on the output characteristics of solitons.

**Fig. 4:**
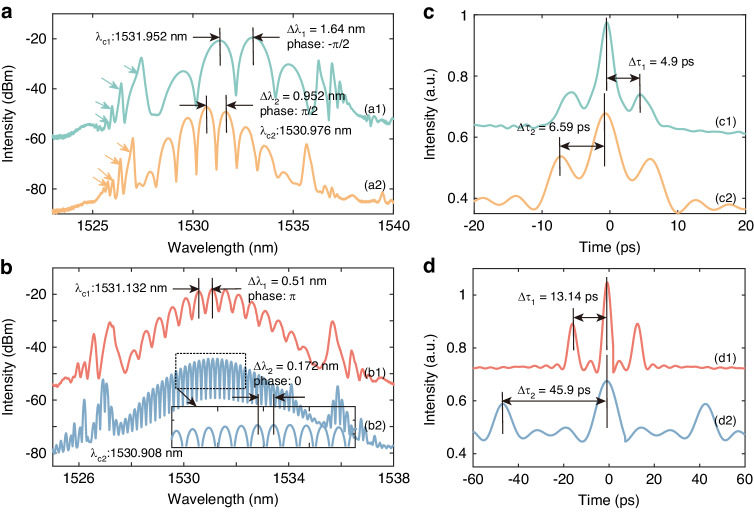
Measured spectra of PHEOD bound soliton pairs with fixed pump power, fine-tuning PC, and taking intra-cavity net *β*_*8*_ as (**a**) –2.66 ps^8^ (green), –3.99 ps^8^ (light orange), (**b**) –5.332 ps^8^ (orange and blue). **c**, **d** Corresponding autocorrelation traces

**Fig. 5:**
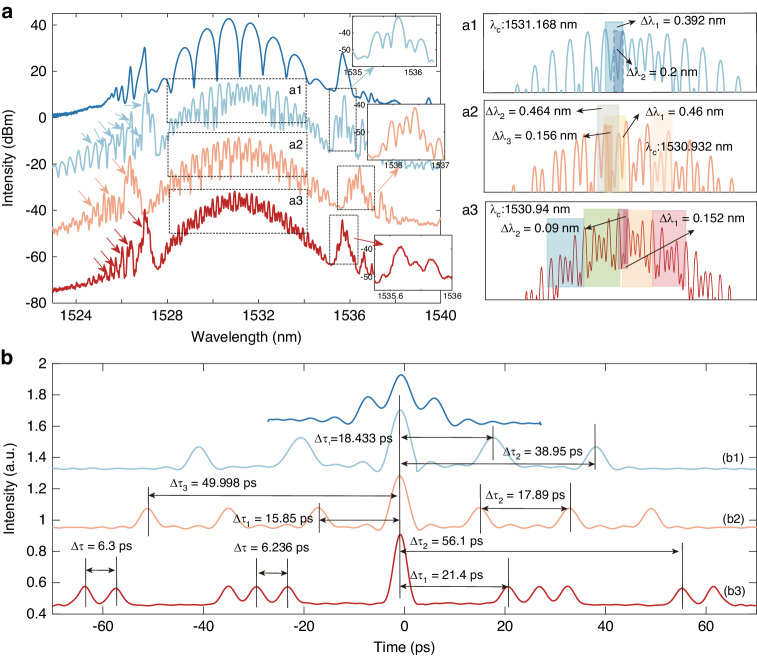
Measured spectra of PHEOD bound multi-solitons with fixed pump power, fine-tuning PC and taking (**a**) *β*_*8*_ as -3.99 ps^8^ (light blue, red), *β*_*10*_ as –3.99 ps^10^ (light orange). **a1a2a3** is the amplified spectra illustrated by the dotted box. **b** Corresponding autocorrelation traces

Bound soliton pairs are the most prevalent form of bound solitons. In general, bound soliton pairs can be categorized into four types based on the phase difference between the two solitons: 0 (in-phase), π (out-of-phase), and ±π∕2^45,46^. Both the 0-phase and π-phase bound soliton pairs exhibit a shared characteristic of an axisymmetric spectrum. However, the spectrum center of a 0-phase bound soliton pair is the smallest, while conversely, that of a π-phase bound soliton pair is the largest. The −π∕2-phase and π∕2-phase bound soliton pairs both exhibit a minimum spectrum center. A distinguishing feature of the −π∕2-phase bound soliton pairs is that the right peak is larger than the left peak in their spectrum, while the π∕2-phase bound soliton pairs display the opposite characteristic. Recognizing the crucial role of phase relationships in forming bound states, we meticulously adjusted the paddle direction of the three-ring PC and the value of intra-cavity net *β*_*8*_ with fixed pump power. This led to the single PHEOD soliton eventually splitting into two PHEOD solitons, which then evolved into the PHEOD bound soliton pair. From traces in Fig. 4a, b, the spectra exhibit regular and pronounced modulation, a typical feature of phase-locked bound solitons. The modulation periods of the spectra (*∆λ*) are related to the pulse separations (*∆τ*), and this specific relationship can be expressed by Eq. (2)^47^:

2 $$\varDelta \tau ={{\lambda }_{0}}^{2}/(c\cdot \varDelta \lambda )$$

where *c* and *λ*_*0*_ are the speed of light in vacuum (3 × 10^8 ^m s^-1^) and the center wavelength, respectively. Table 1 summarizes the parameters of PHEOD bound solitons in Figs. 4, 5. It can be observed that the pulse separation is in inverse proportion to the modulation period, which satisfies Eq. (2).

**Table 1:** Characteristics of PHEOD bound multi-solitons corresponding to Fig. 4 and 5^a^

F. N.	M. P.	C. W.	P. S.	I. R.
4a1c1	1.64 nm	1531.952 nm	4.9016 ps	1:3:1
4a2c2	0.952 nm	1530.976 nm	6.5857 ps	1:1.8:1
4b1d1	0.51 nm	1531.132 nm	13.04738 ps	1:1.9:1
4b2d2	0.172 nm	1530.908 nm	45.948529 ps	1:1.6:1
5a1b1	0.392 nm	1531.168 nm	18.4336 ps	1:1.4:2.5:1.4:1
	0.2 nm		38.95 ps	
5a2b2	0.464 nm	1530.932 nm	15.8498 ps	1:1:1:2.5:1:1:1
	0.46 nm		16.25781 ps	
	0.156 nm		49.9978 ps	
5a3b3	0.152 nm	1530.94 nm	56.1 ps	1:1:1:1:1:3.87:1:1:1:1:1

^a^’*F*. *N*.: figure number; *M*. *P*.: modulation period; *C*. *W*.: central wavelength; *P*. *S*.: pulse separation; *I*. *R*.: intensity ratio of autocorrelation traces.’

Traces (a1) and (a2) exhibit the smallest spectrum centers at 1531.952 nm and 1530.976 nm, respectively, with corresponding modulation periods of ~1.64 nm and ~0.952 nm. Trace (b1) displays a symmetrical structure centered at 1531.132 nm with a modulation period of ~0.51 nm. The corresponding autocorrelation traces (c1), (c2), and (d1) in Fig. 4c, d indicate the pulse separation aligns with the modulation period, with values of ~4.9 ps, ~6.59 ps, and ~13.41 ps, respectively. The pulse separation between two PHEOD solitons is ~1.8, ~1.3, and ~2.8 times the pulse duration, indicating strong interaction between two PHEOD solitons in these three instances. The combination of spectra (a1), (a2), and (b1) with autocorrelation traces (c1), (c2), and (d1) confirms that the phase difference between two PHEOD solitons is approximately -π/2, π/2, and π, indicative of tightly bound states. Due to the bandwidth limitations of oscilloscopes and photodetectors, our oscilloscopes are unable to display such tightly bound solitons. The intensity ratio of the three peaks in autocorrelation traces (c1), (c2), and (d1) are 1:3:1, 1:1.8:1, and 1:1.9:1, respectively, indicating the intensity differences of two PHEOD solitons within bound states. The π-phase emerges from the balanced interaction between repulsions, induced by cross-phase modulation (XPM), and attractions, brought about by spectrum filtering. Specifically, the gain-filtering effect imposed by the gain fiber can cooperate with the XPM effect to culminate in the π-phase bound soliton^48^. Bound solitons are sensitive to changes in laser cavity parameters such as dispersion, nonlinearity, gain, and loss. The adjustment of intra-cavity loss and birefringence can be accomplished by suitably manipulating the PC, thereby facilitating the easy generation of different bound solitons. For instance, in our experiments, PHEOD bound soliton pairs transition from tightly to loosely bound when adjusting the PC with fixed pump power. Trace (b2) presents a symmetric smallest spectrum center at 1530.908 nm with a modulation period of 0.172 nm. The separation between two PHEOD solitons of trace (d2) is 6.3 times the pulse duration, verifying the state as the 0-phase PHEOD loosely bound soliton pair. In addition, the intensity ratio of the autocorrelation trace of this PHEOD loosely bound soliton is 1:1.6:1. It should also be noted that as the pulse separation decreases, the modulation depth of the spectra increases. This suggests that tightly bound soliton pairs exhibit a larger modulation depth on spectra, potentially due to the enhanced interaction between the solitons. Furthermore, spectra in Fig. 4a, b have multi-sidebands (indicated by arrows) induced by large intra-cavity *β*_*8*_, and PHEOD tightly bound soliton pairs with different phases are obtained by changing the intra-cavity net *β*_*8*_, which proves that the phase difference between PHEOD solitons is not only related to the pump power and cavity length^49^ but also the intra-cavity high-even-order dispersion. In this work, self-start mode-locking is mainly operated by SESAM and can maintain stationary mode-locked, while nonlinear polarization rotation (NPR) dominates pulse shaping^50–52^. It enables us to control the interaction between solitons independently by adjusting the direction of the PC to achieve different phases or pulse separation.

The formation of bound solitons can be attributed to the balance of attraction and repulsion between solitons introduced by the soliton-continuum interaction, which is a periodical function with a series of equilibrium points^53^. Previous studies have demonstrated that the number of solitons within the bound state increases with the pump power^54^. However, our experimental results show that the identical phenomenon can be realized through the intra-cavity high-order dispersion management and the adjustment of the PC while maintaining a constant pump power. Figure 5a, b depict the spectra of PHEOD bound tri-soliton, four-soliton, and six-soliton, along with their corresponding autocorrelation traces under different *β*_*8*_. Unlike PHEOD bound soliton pairs, the spectral modulation of PHEOD bound multi-soliton is no longer singular. A distinct secondary modulation (light blue rectangle) can be observed in Fig. 5a1, with modulation periods of 0.392 nm (light blue rectangle) and 0.2 nm (dark blue rectangle). The corresponding pulse separations in trace (b1) of Fig. 5b are 18.433 ps and 38.95 ps. Furthermore, the enlarged spectra in Fig. 5a2 exhibit a distinct cubic modulation (skin rectangle), with modulation periods of 0.46 nm (yellow rectangle), 0.464 nm (gray rectangle), and 0.156 nm (orange rectangle). These correspond to pulse separations of 15.85 ps, 17.89 ps, and 49.998 ps in trace (b2) of Fig. 5b. It is noteworthy that the intensity difference of PHEOD solitons within PHEOD bound multi-solitons results in an intensity ratio of autocorrelation traces close to 1:1.4:2.5:1.4:1 and 1:1:1:2.5:1:1:1. As indicated by the various colored arrows in Fig. 5a, the presence of high-order dispersion causes the PHEOD bound multi-solitons to have multiple sidebands, which also exhibit modulation characteristics (insets in Fig. 5a). These unusual structural bound multi-solitons can be achieved by tuning the PC and intra-cavity high-order dispersion without increasing the pump power^55^. As shown in Fig. 5a3, a sequence of modulated peaks between adjacent maximum peaks indicates the complex interactions among the inner solitons. Its autocorrelation trace comprises two PHEOD bound state units: one unit is a PHEOD bound tri-soliton, and the other unit is a PHEOD bound soliton pair. The intensity ratio of different peaks is close to 1:1:1:1:1:3.87:1:1:1:1:1, suggesting that the unusual structural PHEOD bound multi-soliton may be unstable, with variable pulse intensity and separation^56^. Such unusual structural PHEOD bound multi-solitons further substantiate the possibility of changing the number of PHEOD solitons through high-order dispersion management without adjusting the pump power. We qualitatively validated the experimental results through modeling PHEOD fiber laser and analyzed simulation results in detail in the “Supplementary Information”.

### Simulation results

Numerical modeling of the laser dynamics is a crucial instrument that serves two objectives. First, it facilitates a profound understanding of the physics and operational dynamics inherent to lasers. Second, it offers a straightforward and expedient method for investigating various operational regimes. Given the multitude of variable parameters and the intricacy of laser systems, it is virtually impossible to experimentally explore the entire parameter space, necessitating the usage of simulation. To deepen our comprehension of the experimental observations, we executed numerical simulations of the fiber laser employed in our experiments. This approach allowed us to provide a qualitative explanation for the observed PHEOD bound multi-solitons. Further details regarding the simulation model are elaborated in the “Materials and methods” section.

### Build-up of stationary pure-high-even-order dispersion bound solitons

Bound soliton can be characterized and analyzed at qualitatively various levels of detail. The characteristics of each soliton are described by its modulation period, phase difference, and pulse separation, while the dynamics are characterized by the evolution of separations and relative phases^57^. In general, bound soliton dynamics are characterized based on the shot-to-shot spectrum, that is, the evolution of intensity profiles of intra-cavity fields as a function of RTs, as well as the separations and the relative phases evolution between solitons, which provides a general overview of the way solitons move with each other^57^. Both separations (*τ*) and relative phases (*∆φ*) can be obtained from the interferogram by considering a bound state as a superstition of temporally separated individual solitons. For instance, the field of a bound soliton pair, characterized by the envelopes *E*_*1*_*(t)* and *E*_*2*_*(t)* at frequency *ω*_*0*_, can be expressed as $E(t)=\mathrm{Re}\{[{E}_{1}(t)+{E}_{2}(t)]\exp (i{\omega }_{0}t)\}$. If they have identical envelopes, *E*_*2*_*(t)* can be replaced by${E}_{1}(t+\tau )\exp (i\varDelta \varphi )\}$. The separation *τ* translates to a frequency-domain phase factor *exp(iωτ)*, which modulates the spectrum *E(ω)* with a fringe *1/τ*. Therefore, *τ* is mapped into a modulation observed as an interferogram$S(\omega )=|E(\omega ){|}^{2}$, and the phase of the fringe pattern at *ω*_*0*_ encodes the *∆φ* between the two solitons as $S(\omega )\propto {|{E}_{1}(\omega -{\omega }_{0})|}^{2}[1+\,\cos (\omega \tau -{\omega }_{0}\tau +\varDelta \varphi )]$^20^. As the bound state contains more than two solitons, the information concerning the *∆φ* and *τ* between solitons can be retrieved through the methods of spectrum interferometry under certain conditions^24,58^.

**Fig. 6:**
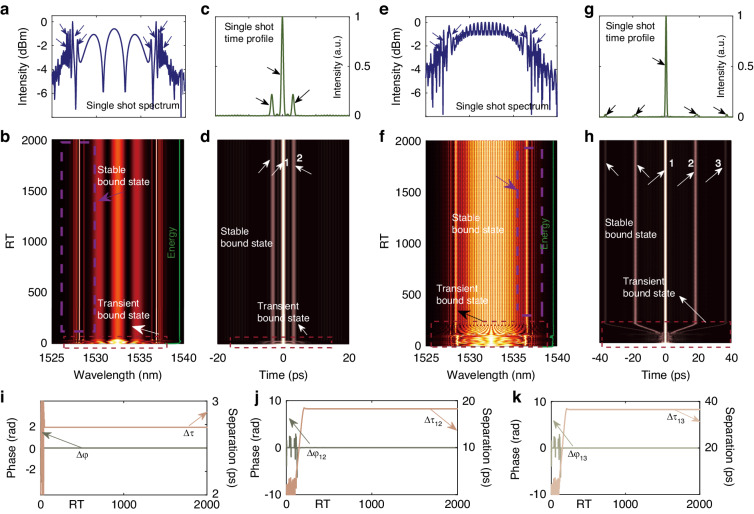
Stationary PHEOD bound soliton pairs (*E*_*sat*_ = 117 pJ, *β*_*8*_ = -6.1 ps^8^) and stationary PHEOD bound tri-soliton (*E*_*sat*_ = 117 pJ, *β*_*8*_ = -9.8 ps^8^). **a**, **e** Single-shot spectrum and (**b**, **f**) shot-to-shot spectrum. **c**, **g** Single-shot autocorrelation trace and (**d**, **h**) filed autocorrelation trace. **i, j**, **k** Corresponding retrieved separation and relative phase of (**b**, **f**)

In other words, various relative phases and separations can be retrieved from the shot-to-shot spectrum, depicting the energy flow between each constituent facilitated by gain dynamics and soliton interactions^59^. Figure 6 provides the build-up of stationary PHEOD bound soliton pair and stationary PHEOD bound tri-soliton. Under the synergistic influence of gain, dispersion, and nonlinear effects, the PHEOD soliton undergoes rapid growth and splits into a transient PHEOD bound soliton before transitioning into a stable PHEOD bound soliton, as indicated by the red dashed rectangles in Fig. 6. The distinct spectral interference patterns in Fig. 6b, f and the near-constant separations and relative phases in Fig. 6ijk suggests their stability. The soliton interactions present within these RTs can be revealed by the first-order (field) autocorrelation trace. According to the Wiener-Khinchin theorem, the Fourier transform of the shot-to-shot spectrum yields the field autocorrelation trace. It should be noted that if the number of solitons is *N* then the corresponding field autocorrelation trace has *2N-1* peaks^44^. The Fourier transforms of the shot-to-shot spectrum in Fig. 6b, f provide field autocorrelation traces in Fig. 6dh. The presence of equally spaced and invariant three peaks (Fig. 6c) or five peaks (Fig. 6g) further substantiates the stability of PHEOD bound multi-solitons. Energy evolution provides an effective approach to comprehending the stationary and non-stationary dynamics of nonlinear systems. We computed the energy evolution by integrating the spectrum across the entire spectral band^19^, as represented by the white curve. The nearly constant energy suggests no energy transfer between PHEOD solitons. In addition, the single-shot spectrum in Fig. 6, e exhibit a series of additional sidebands, beyond Kelly sidebands, induced by *β*_*8*_, as indicated by the purple arrows.

### Dynamics of pure-high-even-order dispersion bound soliton pairs

**Fig. 7:**
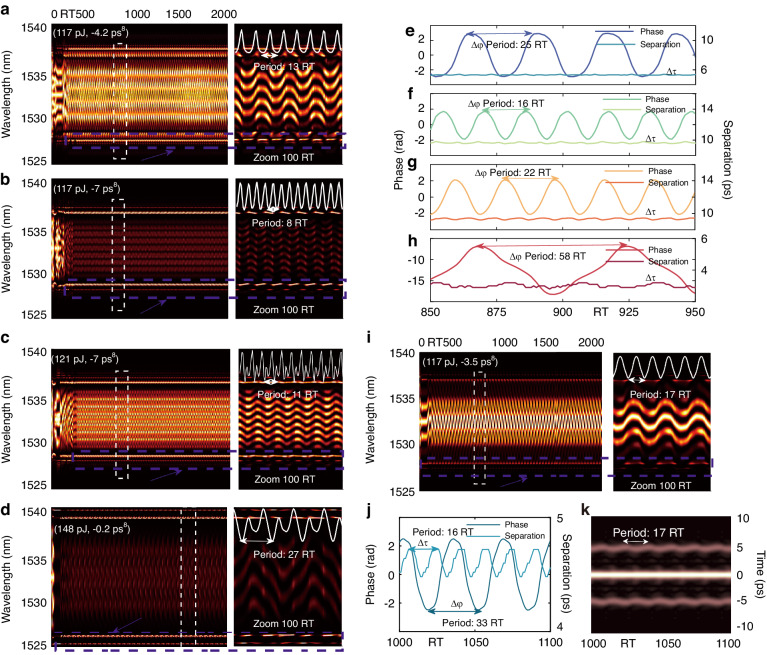
Vibrating phase PHEOD bound soliton pairs. **a–d**, **i** Shot-to-shot spectrum, the right part is enlarged of virtual rectangles. **e–h**, **j** Corresponding retrieved separation and relative phase. **k** Field autocorrelation trace corresponding to dashed rectangles in (**i**)

The experimental results suggest that by adjusting *β*_*8*_ and the direction of PC, PHEOD bound solitons with diverse characteristics can be generated. We investigate PHEOD bound solitons within a two-dimensional parameter space (*E*_*sat*_, *β*_*8*_), where *E*_*sat*_ signifies the intra-cavity loss (power) variation induced by PC, and *β*_*8*_ represents the intra-cavity net eighth-order dispersion. In addition to the stationary PHEOD bound soliton pair with fixed separation and relative phase, we observe vibrating phase PHEOD bound soliton pairs and sliding phase PHEOD bound soliton pairs under different (*E*_*sat*_, *β*_*8*_) conditions. Figure 7 shows the simulation results of vibrating phase PHEOD bound soliton pairs. The periodic evolution of the shot-to-shot spectrum in Fig. 7a–d signifies the presence of periodic interactions between two PHEOD solitons. The zoom-in plot of dashed rectangles further reveals that these four types of PHEOD bound soliton pairs possess distinct oscillation amplitudes and periods. By integrating spectra across the entire spectrum band, we obtained periodic energy evolution curves (white line) with oscillation periods of 13 RTs, 8 RTs, 11 RTs, and 27 RTs. To delve deeper into their characteristics, we calculated the evolution of separations and relative phases based on the shot-to-shot spectrum. The results in Fig. 7e–h demonstrate that separations remain nearly constant during the spectrum variation, while relative phases exhibit periodic oscillations with periods of 25 RTs, 16 RTs, 22 RTs, and 58 RTs, respectively. Phase oscillation implies a weak bond between PHEOD solitons, and the relative intensity between PHEOD solitons undergoes an oscillation process concurrent with the phase oscillation^59,60^.

It is noteworthy that the oscillation period of the relative phase is precisely an integral multiple of the energy evolution period^60^. For instance, as seen in Fig. 7f, the oscillation period of the relative phase is 16 RTs, which is twice the evolution period of energy, which suggests that all parameters of the bound state become self-consistent after 16 RTs, rather than evolving into chaos^60^. Under the condition of (117 pJ, -3.5 ps^8^), we obtained a vibrating PHEOD bound soliton pair^21^. Apart from periodic oscillations of the shot-to-shot spectrum (Fig. 7i), its relative phase and separation also exhibit periodic changes (Fig. 7j). The relative phase oscillation period (33 RTs) is also twice the energy evolution period (17 RTs). Simultaneously, the field autocorrelation trace corresponding to the enlarged part (Fig. 7k) displays the time evolution of the PHEOD bound soliton pair. The minor periodic oscillation indicates the separation vibration, further substantiating the existence of a vibrating PHEOD bound soliton pair. A vibrating bound soliton pair primarily involves several rapid phase oscillations superimposed on a slowly vibrating motion^19^.

**Fig. 8:**
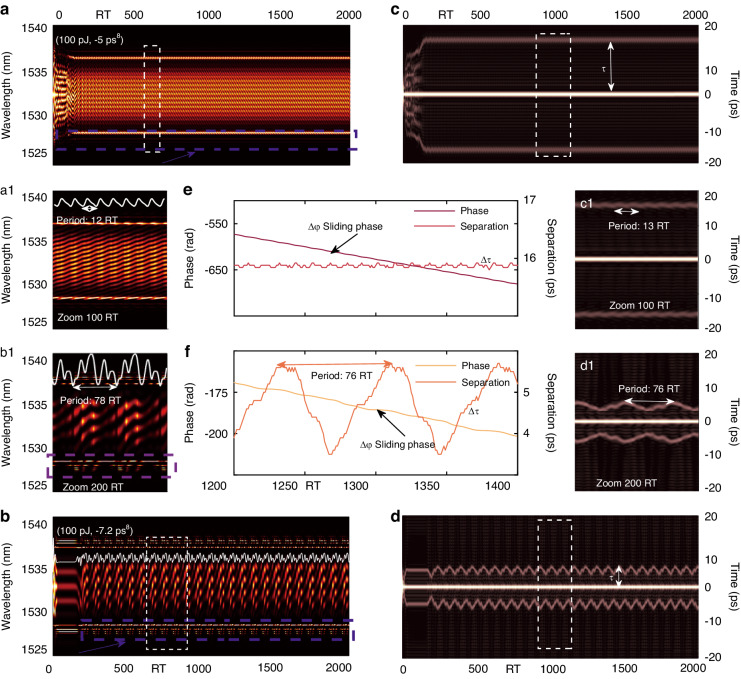
Sliding phase PHEOD bound soliton pairs. **a**, **b** Shot-to-shot spectrum, **a1b1** the enlarged, (**e**, **f**) retrieved separation and relative phase of virtual rectangles in (**a, b**). (**c**, **d**) Field autocorrelation trace, (**c1d1**) enlarged white virtual rectangles

The results in Fig. 7 confirm that *β*_*8*_ significantly influences the spectral and phase evolution characteristics of PHEOD bound soliton pairs. Furthermore, the increase of *E*_*sat*_ leads to more complex energy changes in PHEOD bound soliton pairs. This complexity may be attributed to the intriguing yet intricate nonlinear evolutions of bound soliton pairs, which are primarily governed by gain dynamics, resulting in a rich variation of separation and relative phase between the constituents^19,24^. To investigate whether large *E*_*sat*_ is the sole factor inducing complex energy changes, we reduced *E*_*sat*_ to a low value of 100 pJ. We observed complex energy evolution in sliding phase PHEOD bound soliton pairs by adjusting *β*_*8*_. The shot-to-shot spectrum (Fig. 8a, b) under conditions of (100 pJ, –5 ps^8^) and (100 pJ, –7.2 ps^8^) display rapid and complex changes, with spectral fringes shifting towards longer wavelengths. This shift signifies a large intensity difference between the two PHEOD solitons^61^. The relative phase evolution (Fig. 8e, f), derived from Fig. 8a, b, exhibits sliding characteristics, while the separation evolution demonstrates distinct characteristics. Figure 8e depicts quasi-periodic minor oscillations of separation, whereas Fig. 8f presents a more pronounced periodic oscillation with a period of 76 RTs. Field autocorrelation traces in Fig. 8c, c1 reveal the temporal evolution of the former PHEOD bound soliton pair, and the minor periodic oscillation structure (13 RTs) indicates a minor interaction. In contrast, the detailed field autocorrelation trace of the latter PHEOD bound soliton pair (Fig. 8dd1) exhibits a significant periodic variation (76 RTs), corresponding to periodic attractive and repulsive interactions. The oscillation period of the separation (13 RTs, 76 RTs) closely matches the energy evolution period (12 RTs, 78 RTs). Therefore, these multifaceted internal motions can be attributed to the intricate energy oscillation of each constituent within PHEOD bound soliton pairs^62,63^. The corresponding energy evolution also exhibits a relatively complex periodicity, including four peaks (white curve in Fig. 8b1). These findings suggest that the energy evolution of PHEOD bound soliton pairs can undergo complex periodic changes even at low *E*_*sat*_, and the presence of *β*_*8*_ may induce more complex interactions between PHEOD solitons^14^. Perturbations in fibers, PC, or optical platforms can force stationary bound soliton pairs to transition to such dynamic states in experiments^44^.

### Dynamics of pure-high-even-order dispersion bound tri-soliton

**Fig. 9:**
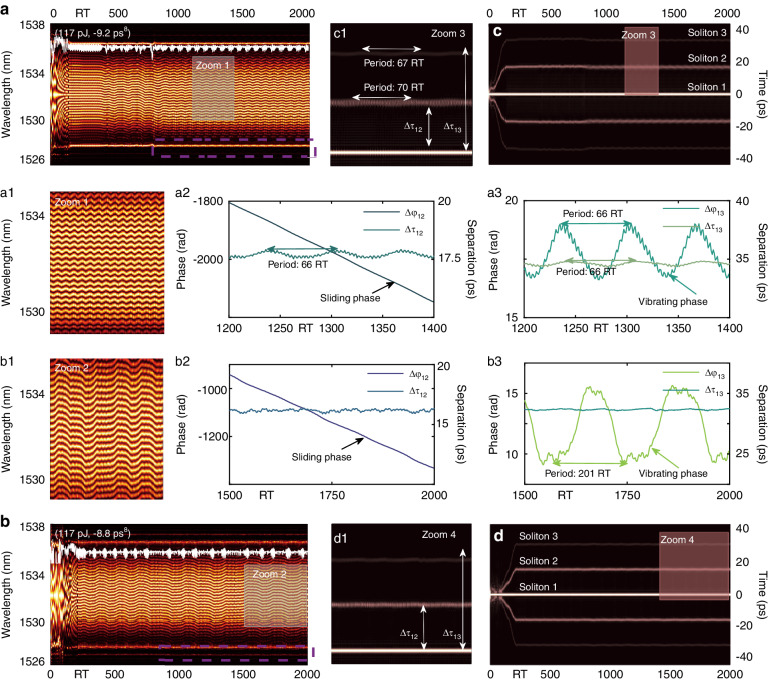
Hybrid phase PHEOD bound tri-solitons. **a**, **b** Shot-to-shot spectrum, **a1b1** enlarged gray rectangles, **a2a3b2b3** corresponding retrieved separation and relative phase. **c**, **d** Field autocorrelation trace, **c1d1** enlarged red rectangles

Experimental results in this work demonstrate that the number of PHEOD solitons within bound states can be increased and the PHEOD bound multi-soliton with different combinations can be achieved by fine-tuning PC or adjusting high-order dispersion at fixed pump power. Simulation results in Figs. 6, 7, 8, 9, and Table 2 validate the above conclusion: PHEOD bound solitons pairs and PHEOD bound tri-solitons with different characteristics can be realized by only adjusting *β*_*8*_. The spectrum interferograms can reveal the relative phases of bound tri-solitons^24,58^. In the presence of mutual interactions with different solitons, the internal dynamics of bound tri-soliton should be much more diverse and involve recurrent motions of different solitons. Guided by PHEOD solitons marked as soliton 1, soliton 2, and soliton 3 of bound tri-solitons, we characterize various PHEOD bound tri-solitons by analyzing the consecutive shot-to-shot spectrum interferograms and their corresponding calculation results, represented by the variables *(∆τ*_*12*_, *∆φ*_*12*_) and (*∆τ*_*13*_, *∆φ*_*13*_). The shot-to-shot spectrum of the PHEOD bound tri-soliton in Fig. 9a exhibits periodic oscillation characteristics similar to vibrating phase PHEOD bound soliton pairs in Fig. 7. The complex energy changes (white curve) suggest the existence of complex energy flow between three PHEOD solitons, which may lead to their complex interactions. We obtained corresponding field autocorrelation traces through the Fourier transform as presented in Fig. 9a2 and a3. Five bright fringes are symmetrically distributed with tiny periodic attraction and repulsion between three PHEOD solitons. Fig. 9a2 and a3 display the separations and relative phases retrieved from Fig. 9a1. Figure 9a2 shows that soliton 1 and soliton 2 form a PHEOD bound soliton pair with sliding phase and oscillating separation, while Fig. 9a3 shows that soliton 1 and soliton 3 form a vibrating PHEOD bound soliton pair where the relative phase and separation oscillate simultaneously. Particularly, for conventional bound solitons, it has been reported that oscillating phases and oscillating separations result from the periodic variation of pulse intensities within bound soliton pairs, while the sliding phase is governed by the persistent intensity difference between each constituent, further regulating the oscillatory motions^19^. By adjusting the parameter *β*_*8*_, we observed another type of PHEOD bound tri-soliton. This variant exhibited virtually invariant separations and demonstrated both sliding and oscillating phase characteristics. The shot-to-shot spectrum, relative phase evolution, and separation evolution in Fig. 9bb1b2b3 demonstrates the existence of such PHEOD bound tri-soliton. Its field autocorrelation trace is present in Fig. 9d, and Fig. 9d1 is the zoom-in plot of the red rectangle, which proves that there is a significant intensity difference between the three PHEOD solitons, but there is almost no interaction between them, resulting in the separation between the PHEOD solitons being unchanged, as verified by the equally-spaced straight five peaks. However, the energy evolution curve in Fig. 9b indicates the existence of complex energy exchange between three PHEOD solitons. Therefore, for PHEOD bound solitons, the phase evolution is not only related to the intensity difference between PHEOD solitons but also closely related to the energy exchange between PHEOD solitons.

**Table 2:** Detailed characteristics of PHEOD bound multi-solitons corresponding to Figs. 6–9

F. N.	Parameters	Soliton number	Separation evolution	Phase evolution
6b	(117 pJ, –6.1 ps^8^)	2	Stationary	Stationary
6 f	(117 pJ, –9.8 ps^8^)	3	Stationary	Stationary
7a	(117 pJ, –4.2 ps^8^)	2	Stationary	Vibrating
7b	(117 pJ, –7 ps^8^)	2	Stationary	Vibrating
7c	(121 pJ, –7 ps^8^)	2	Stationary	Vibrating
7d	(148 pJ, –0.2 ps^8^)	2	Stationary	Vibrating
7i	(117 pJ, –3.5 ps^8^)	2	Stationary	Vibrating
8a	(100 pJ, –5 ps^8^)	2	Stationary	Sliding
8b	(100 pJ, –7.2 ps^8^)	2	Vibrating	Sliding
9a	(117 pJ, –9.2 ps^8^)	3	Vibrating	Sliding and vibrating
9b	(117 pJ, –8.8 ps^8^)	3	Stationary	Sliding and vibrating

## Discussion

Based on our experimental and simulation results, we found that fiber lasers with high-order dispersion management and varying cavity parameters can converge to different attractors. These attractors correspond to various emission states, ranging from PHEOD single soliton to diverse PHEOD bound multi-solitons. The high-even-order dispersion, akin to the second-order dispersion for conventional solitons, plays a significant role in the study of PHEOD solitons and their bound states. Compared with conventional solitons, PHEOD solitons present similar but unique characteristics when considering the linear and nonlinear interplay in fiber lasers. Upon generation, multi-solitons evolve into various states, influenced by the interactions between the solitons and intra-cavity dispersive waves. Typically, multi-solitons are distributed randomly within the cavity. However, by tuning the pump power and adjusting the paddle direction of the PC, they can self-organize and position themselves within the cavity at different separations. The coherent solitons will bind together to form various bound states as the phase difference of these solitons remains constant. Similarly, PHEOD solitons will split once the high-even-order dispersion fails to balance the nonlinear, and by tuning the direction of PC and changing high-even-order dispersion, they can form various PHEOD bound states. Increasing the pump power is an effective method to augment the number of intra-cavity conventional solitons. Nevertheless, experiment and simulation results in this paper suggest that the number of PHEOD solitons can be increased by adjusting the intra-cavity high-even-order dispersion, even while maintaining a constant pump power.

The formation of bound solitons is primarily due to soliton interactions, which can be categorized into three types: short-range, long-range, and global interactions^41^. When the separation between solitons is close, the direct overlap of pulse tails causes short-range interactions that can be attractive or repulsive, depending on the relative phase difference between solitons. The strength of these interactions decreases exponentially with increasing separation. The long-range interaction is induced by the acoustic response dominated by the electrostrictive effects in fibers. It is known that dispersive waves can cause long-range repulsive interactions in mode-locked fiber lasers. The global interactions originate from the intra-cavity unstable continuous wave. These three types of soliton interactions have distinct interaction conditions and can coexist or exist independently. By manipulating various parameters within fiber lasers, a balance among the three types of interactions can be achieved, thereby facilitating the formation of different types of bound solitons. In ref. ^40^, it was predicted that the overlapping of oscillatory tails gives rise to potential minima which account for the generation of bound solitons. This prediction was experimentally confirmed in micro-resonator Kerr cavities, where intra-cavity leading solitons with tails appear, and newly generated solitons evolve from such oscillatory tails^39^. Since the group velocity of the oscillatory tails and solitons differs, the position of the newly generated solitons automatically adjusts under the effect of nonlinearity and intra-cavity dispersion, eventually forming bound solitons^39^. The temporal oscillatory tail is a characteristic feature of PHEOD solitons^14,16^. In ref. ^36^, it was found that the enhanced interaction caused by large intra-cavity β_4_ can promote the transformation of quasi-periodical pulsating pure-quartic bound solitons into periodical pulsating. This implies that a periodic energy exchange between two solitons can be facilitated by the overlap of their oscillatory tails^33,41,42^. Furthermore, there may be more possibilities for energy exchange within the PHEOD bound multi-soliton, leading to their distinct characteristics.

In the context of mode-locked fiber lasers, Kelly sidebands are produced as a result of the interaction between solitons and dispersive waves. This interaction occurs when the wavelength of the dispersive waves aligns with the phase-matching condition. Consequently, these dispersive waves present themselves as Kelly sidebands, which invariably share intra-cavity energy with solitons^53^. Studies have confirmed that these Kelly sidebands, which correspond to temporal oscillatory tails, play a crucial role in the generation of bound solitons within mode-locked fiber lasers^43,44^ and oscillate in sync with pulsating bound soliton pairs, suggesting that the energy transfer between sidebands and solitons could result in various bound states^59^. The spectrum of PHEOD solitons exhibits a series of sidebands induced by high-even-order dispersion. Our simulation findings suggest that by manipulating the high-even-order dispersion, we can generate PHEOD bound solitons exhibiting different characteristics. The shot-to-shot spectrum of these PHEOD bound solitons undergoes alterations in sidebands, as indicated by the purple dashed rectangles in Figs. 7–9. Concurrently, the corresponding field autocorrelation traces display oscillatory behavior. Therefore, we hypothesize that the temporal oscillatory tails and multi-sidebands induced by high-even-order dispersion could be the underlying mechanism for obtaining PHEOD bound solitons with different soliton numbers and characteristics, even when the pump power remains constant.

In conclusion, we realize the intra-cavity high-order dispersion management based on the spatial light modulator in experiments and obtain pure-quartic, -sextic, -octic, and -decic solitons. Under the condition of fixed pump power, by adjusting high-even-order dispersion and the paddle direction of the polarization controller, we obtained the PHEOD bound solitons with varying numbers of solitons, separations, and combinations. Subsequent simulations were conducted to validate our experimental results. Further analysis was carried out on the formation of stationary PHEOD bound multi-solitons, the characteristics of vibrating phase PHEOD bound soliton pairs, sliding phase PHEOD bound soliton pairs, and hybrid phase PHEOD bound tri-solitons. This work contributes additional insights into the complex dynamics of PHEOD bound solitons, enhancing our understanding of this phenomenon.

## Materials and methods

### Experimental setup and measurement system

The PHEOD soliton passively mode-locked fiber laser, as depicted schematically in Fig. 1a, comprises four key components: gain, saturable absorber, polarization/loss control, and spectral pulse shaping. A 1.2 m segment of erbium-doped fiber (EDF, SM-ESF-7/125) is pumped by a 976 nm laser source via a 980/1550 nm wavelength division multiplexer (WDM) to provide the required gain and the output coupler (OC) extracting 50% of the power from the fiber cavity. Soliton spectrums were recorded by the spectral analyzer (OSA, YOKOGAWA, AQ6370D), and the corresponding autocorrelation traces were measured by a commercial autocorrelator (APE, PulseCheck). SESAM is used to achieve passive mode-locking and employ a three-ring polarization controller (PC) to adjust the intra-cavity loss. The pigtail of the WDM is HI1060, while the pigtails of other intra-cavity devices are SMF28e. The total fiber length of the ring cavity is 26.6 m, corresponding to a repetition rate of 7.9475 MHz. In the absence of a nonlinear effect, the impact of dispersion is equivalent to applying a phase transformation to the intra-cavity field in the spectral domain^16^:

3 $$\tilde{A}(L,\omega )=\tilde{A}(0,\omega ){e}^{i\phi (\omega )}$$

where $\tilde{A}$ is the Fourier transform of the envelope *A*, *ϕ(ω)* represents the dispersion-induced phase and L is the propagation length.

In conventional optical waveguides, the intrinsic dispersion is primarily governed by *β*_*2*_, while the effects of high-order dispersion are typically minimal. To achieve high-order dispersion management, a more flexible technique is required. This technique involves the use of a spectral pulse shaping structure based on the advanced Liquid Crystal on Silicon (LCoS). The spectral pulse shaping structure operates by splitting the constituent wavelengths of pulses into distinct spatial channels, each of which undergoes phase and/or amplitude modulation before being recombined. For instance, the process of wavelength splitting can be executed using a diffraction grating. Following this, a spatial light modulator (SLM) is employed to apply distinct phases and amplitudes to each wavelength. Finally, the pulses are recombined using the same diffraction grating. Specifically, the SLM is placed on the Fourier plane of a 4 f system. According to the Fourier transform of the lens, the time domain information is transformed to the spatial frequency domain, and then restored to the time domain after spatial filtering or spectral modulation, thereby achieving pulse phase adjustment, that is, high-order dispersion management which enables the adjustment of all-order-dispersion. Spectral pulse shaping can be straightforwardly implemented in a fiber laser cavity. By applying the spectrum phase profile *ϕ(ω)*, as depicted in Fig. 1b, we can compensate for the inherent *β*_*2*_ and *β*_*3*_ of the fiber cavity and, at the same time, manage the large negative high-even-order dispersion. The applied phase profile can be expressed as^14^:

4 $$\phi (\omega )=\mathop{\sum }\limits_{n=2}^{3}\frac{{\beta }_{{\rm{n}}}{(\omega -{\omega }_{0})}^{n}}{n!}+\frac{{\beta }_{k}{(\omega -{\omega }_{0})}^{k}}{k!}$$

where *β*_*n*_ is the *n*-th order dispersion for *n* = 2, 3. For the results presented in this work, the dispersion parameter of EDF, HI1060, and SMF28e is -46.25 ps (nm•km)^-1^, 0.059 ps (nm•km)^-1^, and 17 ps (nm•km)^-1^, respectively. The intra-cavity net *β*_*2*_ was calculated to be −0.48 ps^2^. The intra-cavity net *β*_*2*_ was calculated to be −0.48 ps^2^. Therefore, the *β*_*2*_ and *β*_*3*_ loaded in the SLM are set to 0.48 ps^2^ and -0.00012 ps^3^ respectively to compensate for the intra-cavity *β*_*2*_ and *β*_*3*_, where *β*_*3*_ is based on the value reported in refs. ^13,16^. The second term on the right-hand side of Eq. (4) corresponds to the negative high-even-order dispersion required for the generation of pure-quartic (*k* = 4), -sextic (*k* = 6), -octic (*k* = 8), or -decic (*k* = 10) solitons. We analyzed PHEOD solitons and their bound states in the experiment by altering the intra-cavity net high-even-order dispersion (using the above method) and fine-tuning the PC.

### Simulation setup

Numerical modeling of laser dynamics serves two primary objectives. First, it provides valuable insights into the physics and operational dynamics of the lasers. Second, it offers a straightforward and rapid tool for investigating various operating regimes. Due to the large number of variable parameters and the complexity of laser systems, it is virtually impossible to experimentally explore the full parameter space. Therefore, simulations are indispensable. Successful computations necessitate a model that accurately captures the complex dynamics of realistic experiments with minimal approximations. In this paper, we employed the nonlinear Schrödinger equation to describe pulse propagation through each fiber segment:

5 $$\frac{\partial A}{\partial z}=i\sum _{l}\frac{{\beta }_{l}}{l!}{\left(i\frac{\partial }{T}\right)}^{l}A+\frac{g-a}{2}+\frac{g}{2{\varOmega }^{2}}\frac{{\partial }^{2}A}{\partial {T}^{2}}+i\gamma {|A|}^{2}A$$

here, *z* and *T* are the propagation distance and local time; *γ* is the Kerr nonlinear parameter; α represents the linear loss. *Ω* is the 3 dB bandwidth of the gain fiber (doped-fiber), $g={g}_{0}\exp (-\frac{\int ({|A|}^{2})dt}{{E}_{sat}})$ is the gain of fibers, where *g*_*0*_ is the small-signal gain, which is taken to be non-zero only in the intra-cavity gain fiber (doped-fiber), and the saturation energy *E*_*sat*_ can be adjusted to simulate changes in the pump power and the intra-cavity loss. Considering the finite gain bandwidth of EDF, we added a Lorentzian profile filter with a bandwidth of 25 nm to the gain model. The saturable absorber is represented by the transmission function of the intensity $T={\alpha }_{0}-\alpha /(1+\frac{{|A|}^{2}}{{P}_{sat}})$, where *α*_*0*_ is saturation absorption, *α* presents the modulation depth of a saturable absorber, and *P*_*sat*_ is saturable power. The simulation parameters are consistent with their experimental values, that is, 0.45, 0.17, and 25 W. In the simulation, the laser initially propagates through the pigtail fiber (HI1060) of WDM. Subsequently, the intra-cavity solitons are amplified by the 1.2 m EDF due to its saturable amplification property and exhibit an almost linear increase in the initial part, while the enlargement rate slows down in the latter part. Following the EDF, the solitons traverse through the SMF28e and are outputted by a 50% output coupler. Then, the solitons propagate further through the circulator and the SESAM, where the duration and intensity decrease due to the saturable absorption effect. In the final section, spectral pulse shaping is modeled by multiplying the electric field by a phase following the expression in Eq. (4) in the spectrum domain to realize high-order dispersion management.

### Supplementary information

Supplementary Information for Pure-high-even-order dispersion bound solitons complexes in ultra-fast fiber lasers

## Data Availability

The data that support the simulations within this paper are available from the corresponding authors upon reasonable request.

## References

[1] Song Y (2019). Recent progress of study on optical solitons in fiber lasers. Appl. Phys. Rev..

[2] Han Y (2020). Generation, optimization, and application of ultrashort femtosecond pulse in mode-locked fiber lasers. Prog. Quantum Electron..

[3] Turitsyn SK, Bale BG, Fedoruk MP (2012). Dispersion-managed solitons in fibre systems and lasers. Phys. Rep..

[4] Kodama Y (1994). Role of third-order dispersion on soliton instabilities and interactions in optical fibers. Opt. Lett..

[5] Chan KC, Liu HF (1994). Effect of third-order dispersion on soliton-effect pulse compression. Opt. Lett..

[6] Dennis ML, Duling IN (1994). Third-order dispersion in femtosecond fiber lasers. Opt. Lett..

[7] Blanco-Redondo A (2016). Pure-quartic solitons. Nat. Commun..

[8] Lo CW (2018). Analysis and design of fibers for pure-quartic solitons. Opt. Express.

[9] Tam KKK (2020). Generalized dispersion Kerr solitons. Phys. Rev. A.

[10] Wang ZT (2021). An exact soliton-like solution of cubic-quintic nonlinear Schrödinger equation with pure fourth order dispersion. Results Phys..

[11] Han Y (2022). Analysis of various soliton pulsation spectro-temporal dynamics in anomalous dispersion fiber laser. Opt. Laser Technol..

[12] Tam KKK (2019). Stationary and dynamical properties of pure-quartic solitons. Opt. Lett..

[13] Runge AFJ (2020). The pure-quartic soliton laser. Nat. Photonics.

[14] Runge AFJ (2021). Infinite hierarchy of solitons: Interaction of Kerr nonlinearity with even orders of dispersion. Phys. Rev. Res..

[15] Sakaguchi H, Skryabin DV, Malomed BA (2018). Stationary and oscillatory bound states of dissipative solitons created by third-order dispersion. Opt. Lett..

[16] De Sterke CM (2021). Pure-quartic solitons and their generalizations—Theory and experiments. APL Photonics.

[17] Gui LL (2018). Soliton molecules and multisoliton states in ultrafast fibre lasers: intrinsic complexes in dissipative systems. Appl. Sci..

[18] Wang ZQ (2023). Spectral pulsations of dissipative solitons in ultrafast fiber lasers: period doubling and beyond. Laser Photonics Rev..

[19] Xia R (2020). Experimental observation of shaking soliton molecules in a dispersion-managed fiber laser. Opt. Lett..

[20] Herink G (2017). Real-time spectral interferometry probes the internal dynamics of femtosecond soliton molecules. Science.

[21] Igbonacho J (2019). Dynamics of distorted and undistorted soliton molecules in a mode-locked fiber laser. Phys. Rev. A.

[22] Lau KY (2023). Real‐time investigation of ultrafast dynamics through time‐stretched dispersive fourier transform in mode‐locked fiber lasers. Laser Photonics Rev..

[23] Wang YZ (2020). Recent advances in real-time spectrum measurement of soliton dynamics by dispersive Fourier transformation. Rep. Prog. Phys..

[24] Xia R (2023). Investigations on diverse dynamics of soliton triplets in mode-locked fiber lasers. Opt. Expr..

[25] Stratmann M, Pagel T, Mitschke F (2005). Experimental observation of temporal soliton molecules. Phys. Rev. Lett..

[26] Kurtz F, Ropers C, Herink G (2020). Resonant excitation and all-optical switching of femtosecond soliton molecules. Nat. Photonics.

[27] Pang M (2016). All-optical bit storage in a fibre laser by optomechanically bound states of solitons. Nat. Photonics.

[28] Jang JK (2015). Temporal tweezing of light through the trapping and manipulation of temporal cavity solitons. Nat. Commun..

[29] Liu YS (2023). Phase-tailored assembly and encoding of dissipative soliton molecules. Light Sci. Appl..

[30] Liu S (2022). On-demand harnessing of photonic soliton molecules. Optica.

[31] Liu XY, Zhang HX, Liu WJ (2022). The dynamic characteristics of pure-quartic solitons and soliton molecules. Appl. Math. Model..

[32] Zhao KJ (2021). Vector quartic solitons in birefringent fibers. Opt. Lett..

[33] Zeng JL (2022). Theory for the interaction of pure-quartic solitons. Appl. Math. Lett..

[34] Zhang ZX (2022). Pulsating dynamics in a pure-quartic soliton fiber laser. Opt. Lett..

[35] Han Y (2023). Creeping and erupting dynamics in a pure-quartic soliton fiber laser. Opt. Expr..

[36] Yang S (2023). Internal motion within pulsating pure-quartic soliton molecules in a fiber laser. Chaos, Solitons Fractals.

[37] Olivier M, Piché M (2009). Origin of the bound states of pulses in the stretched-pulse fiber laser. Opt. Express.

[38] Tang DY (2005). Soliton interaction in a fiber ring laser. Phys. Rev. E.

[39] Xiao ZY (2023). Near-zero-dispersion soliton and broadband modulational instability Kerr microcombs in anomalous dispersion. Light Sci. Appl..

[40] Malomed BA (1991). Bound solitons in the nonlinear Schrödinger-Ginzburg-Landau equation. Phys. Rev. A.

[41] Dai JX (2022). The bound states of pure-quartic solitons. Chaos Solitons Fractals.

[42] Akhmediev NN, Buryak AV (1995). Interactions of solitons with oscillating tails. Opt. Commun..

[43] Soto-Crespo JM (2003). Quantized separations of phase-locked soliton pairs in fiber lasers. Opt. Lett..

[44] Peng JS, Zeng HP (2018). Build-up of dissipative optical soliton molecules via diverse soliton interactions. Laser Photonics Rev..

[45] Gui LL, Xiao XS, Yang CX (2013). Observation of various bound solitons in a carbon-nanotube-based erbium fiber laser. J. Opt. Soc. Am. B.

[46] Komarov A, Komarov K, Sanchez F (2009). Quantization of binding energy of structural solitons in passive mode-locked fiber lasers. Phys. Rev. A.

[47] Chen ZK, Zhou J, Zhao JQ (2023). Switchable and reciprocal soliton bound states enabled by continuously tunable local modal-birefringence in a mode-locked fiber laser. IEEE J. Quantum Electron..

[48] He WB (2021). Synthesis and dissociation of soliton molecules in parallel optical-soliton reactors. Light Sci. Appl..

[49] Zhang XB (2022). Spatiotemporal self-mode-locked operation in a compact partial multimode Er-doped fiber laser. Opt. Lett..

[50] Kim S (2012). Hybrid mode-locked Er-doped fiber femtosecond oscillator with 156 mW output power. Opt. Expr..

[51] Li GL (2023). Modelling the sub-100fs Dy^3+^: Fluoride fiber laser beyond 3 μm. Opt. Laser Technol..

[52] Li X, Zou WW, Chen JP (2015). Passive harmonic hybrid mode-locked fiber laser with extremely broad spectrum. Opt. Expr..

[53] Zhu TY (2019). Observation of controllable tightly and loosely bound solitons with an all-fiber saturable absorber. Photonics Res..

[54] Guo TG (2022). Observation of complex multimode soliton molecules in spatiotemporal mode-locked Er-doped fiber laser. Opt. Commun..

[55] Song YF (2016). Coexistence and interaction of vector and bound vector solitons in a dispersion-managed fiber laser mode locked by graphene. Opt. Express.

[56] Zhang D (2022). SnS_2_ microsheets for optical supramolecular generation. Ann. Der Phys..

[57] Wang ZQ (2019). Optical soliton molecular complexes in a passively mode-locked fibre laser. Nat. Commun..

[58] Luo YY (2020). Real-time dynamics of soliton triplets in fiber lasers. Photonics Res..

[59] Wang QB (2023). Observation of the “invisible” pulsation of soliton molecules in a bidirectional ultrafast fiber laser. Opt. Express.

[60] Wei ZW (2018). Pulsating soliton with chaotic behavior in a fiber laser. Opt. Lett..

[61] Liu RM (2023). Collision-induced Hopf-type bifurcation reversible transitions in a dual-wavelength femtosecond fiber laser. Opt. Express.

[62] Soto-Crespo JM (2007). Soliton complexes in dissipative systems: Vibrating, shaking, and mixed soliton pairs. Phys. Rev. E.

[63] Zhou Y (2022). Dynamics of dissipative soliton molecules in a dual-wavelength ultrafast fiber laser. Opt. Expr..

