# Supplementary material for: Pure-high-even-order dispersion bound solitons complexes in ultra-fast fiber lasers
Source: Light Sci Appl. 2024 May 6;13:101. doi: 10.1038/s41377-024-01451-z (PMC11070426; doi:10.1038/s41377-024-01451-z)
Supplement: Supplementary file 1 — Supplementary Information for Pure-high-even-order dispersion bound solitons complexes in ultra-fast fiber lasers [file 41377_2024_1451_MOESM1_ESM.docx]

# Supplementary Information for

# Pure-high-even-order dispersion bound solitons complexes in ultra-fast fiber lasers

*Ying Han^1^, Bo Gao^1*^, Honglin Wen^1^, Chunyang Ma^2,3^, Jiayu Huo^1^, Yingying Li^1^, Luyao Zhou^1^, Ge Wu^4*^, and Liu Lie^1^*

*^1^College of Communication Engineering, Jilin University, Changchun 130012, China*

*^2^Research Center of Circuits and Systems, Peng Cheng Laboratory, Shenzhen 518055, China*

*^3^International Collaborative Laboratory of 2D Materials for Optoelectronics Science and Technology of Ministry of Education, Institute of Microscale Optoelectronics, Shenzhen University, Shenzhen 518060, China*

*^4^College of Electronic Science and Engineering, Jilin University, Changchun 130012, China*

**Corresponding author. E-mail: Bo Gao: gaobo0312@jlu.edu.cn*

*Ge Wu: wuge@jlu.edu.cn*

**Section 1: Simulation results on pure-high-even-order-dispersion (PHEOD) bound solitons**

As described in the experiment of the manuscript, we found that while keeping the pump power and the blade direction of the polarization controller (PC) unchanged, there will be a modulated spectra by changing the high-even-order dispersion value applied in the spectral pulse shaping structure. We verified the experimental results through theoretical modeling of the fiber laser used in the experiment, as shown in Fig. S1 and Fig. S2. It should be noted that the adjustment of the PC in the experiment can change the intra-cavity power and intra-cavity loss. Therefore, the adjustment of the PC is qualitatively represented by changing the saturation power (*E_sat_*) in the simulation. Table S1 summarizes the parameters of pure-high-even-order dispersion (PHEOD) bound soliton pairs and PHEOD bound multi-soliton, as well as the *E_sat_* and eight-order dispersion (*β_8_*) values used in Figs. S1 and S2.

Bound soliton pairs are the most prevalent form of bound solitons^1-4^. In general, bound soliton pairs can be categorized into four types based on the phase difference between the two solitons: 0 (in-phase), π (out-of-phase), and ±π∕2^4, 5^. The modulation periods of the spectra *(∆λ*) are related to the pulse separations (∆τ), and this specific relationship can be expressed by , where c and λ_0_ are the speed of light in vacuum (3×10^8^ m s^-1^) and the center wavelength, respectively^6^. Spectra curves in Fig. S1ab present regular modulation, which is a typical feature of bound states. Spectrum (a1) and (a2) exhibit the smallest spectrum centers at 1531.49 nm and 1532.82 nm, respectively, with corresponding modulation periods of ~1.76 nm and ~1.37 nm. Trace (b1) and (b2) display a symmetrical structure centered at 1531.13 nm and 1532 nm with a modulation period of ~0.508 nm and ~0.392 nm. The corresponding autocorrelation traces in Fig. S1cd indicate the pulse separation aligns with the modulation period, with values of ~4.48 ps, ~5.8 ps, ~15.72 ps, and ~19.64 ps, respectively. The pulse separation between two PHEOD solitons in Fig. S1c is within 5 times the pulse duration, indicating a strong interaction between two PHEOD solitons, while in Fig. S1d is greater than 10 times, indicating a weak interaction between two PHEOD solitons^7^. The combination of spectra with autocorrelation traces confirms that the phase difference between two PHEOD solitons is approximately -π/2 (a1), π/2 (a2), π (b1), and 0 (b2), manifested as tightly bound state (c1)(c2) and loosely bound state (d1)(d2). The intensity ratio of the three peaks in Fig. S1bc are 1:3.8:1, 1:5:1, 1:7:1, and 1:4.5:1, respectively, indicating the intensity differences of two PHEOD solitons within bound states. It should also be noted that as the pulse separation decreases, the modulation depth of the spectra increases. This suggests that tightly bound soliton pairs exhibit a larger modulation depth on spectra, potentially due to the enhanced interaction between the solitons. PHEOD tightly bound soliton pairs with different phases are obtained by changing the intra-cavity net *β_8_*, which proves that the phase difference between PHEOD solitons is not only related to the pump power and cavity length^8^ but also the intra-cavity high-even-order dispersion.


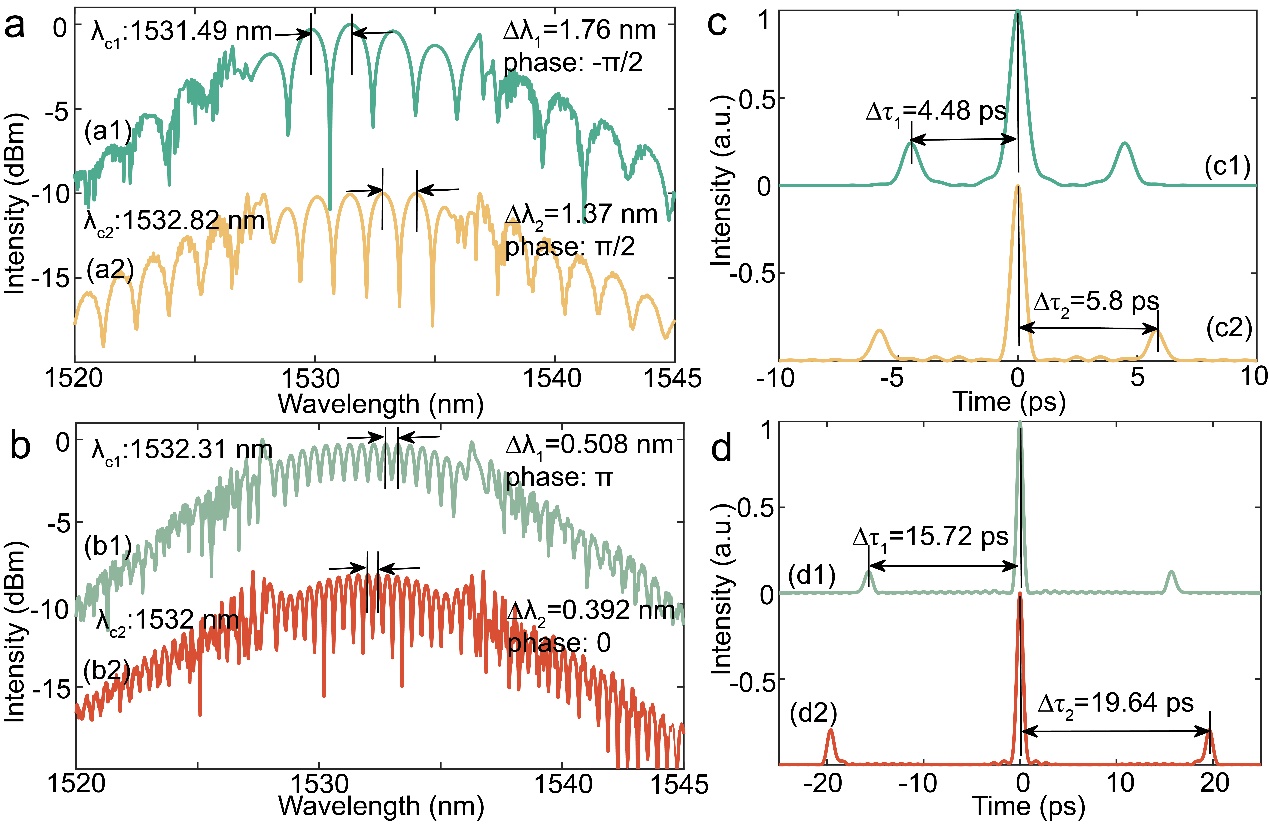


**Fig. S1** Simulation spectra of PHEOD bound soliton pairs taking intra-cavity net β_8_ as **a** -3.5 ps^8^ (green), -7.6 ps^8^ (light orange), **b** -5 ps^8^ (light green and orange). **cd** Corresponding autocorrelation traces.

**Table S1. Characteristics of PHEOD bound multi-solitons corresponding to Fig. S1 and S2^a^**

| **F. N.** | ***E_sat_* (pJ)** | ***β_8_*(ps^8^)** | **C. W. (nm)** | **M. P. (nm)** | **P. S. (ps)** | **I. R.** |
| --- | --- | --- | --- | --- | --- | --- |
| S1a1c1 | 117 | -3.5 | 1531.49 | 1.76 | 4.48 | 1:3.8:1 |
| S1a2c2 | 117 | -7.6 | 1532.82 | 1.37 | 5.8 | 1:5:1 |
| S1b1d1 | 117 | -5 | 1532.31 | 0.508 | 15.72 | 1:7:1 |
| S1b2d2 | 148 | -5 | 1532 | 0.392 | 19.64 | 1:4.5:1 |
| S2af | 117 | -0.4 | 1532.43 | 0.92 | 8.52 | 1:3.5:8:3.5:1 |
|  |  |  |  | 0.431 | 17 |  |
| S2bg | 117 | -9.9 | 1531.64 | 0.4838 | 10.56 | 1:0.9:0.9:8:0.9:0.9:1 |
|  |  |  |  | 0.2365 | 21.6 |  |
|  |  |  |  | 0.1586 | 32.2 |  |
| S2ch | 117 | -0.6 | 1531.76 | 0.392 | 19.1 | 1:0.9:0.9:7:0.9:0.9:1 |
| S2ei | 162 | -9.9 | 1531.29 | 0.313 | 24.48 | 1:1:0.9:8:0.9:1:1 |
| S2fj | 148 | -9.9 | 1531.96 | - | - | 1:0.8:1:0.9:0.9:0.7:12:0.7:0.9:0.9:1:0.8:1 |

^a^‘F. N.: figure number; *E_sat_*: saturation power; *β_8_*: eight-order dispersion; M. P.: modulation period; C. W.: central wavelength; P. S.: pulse separation; I. R.: intensity ratio of autocorrelation traces.’


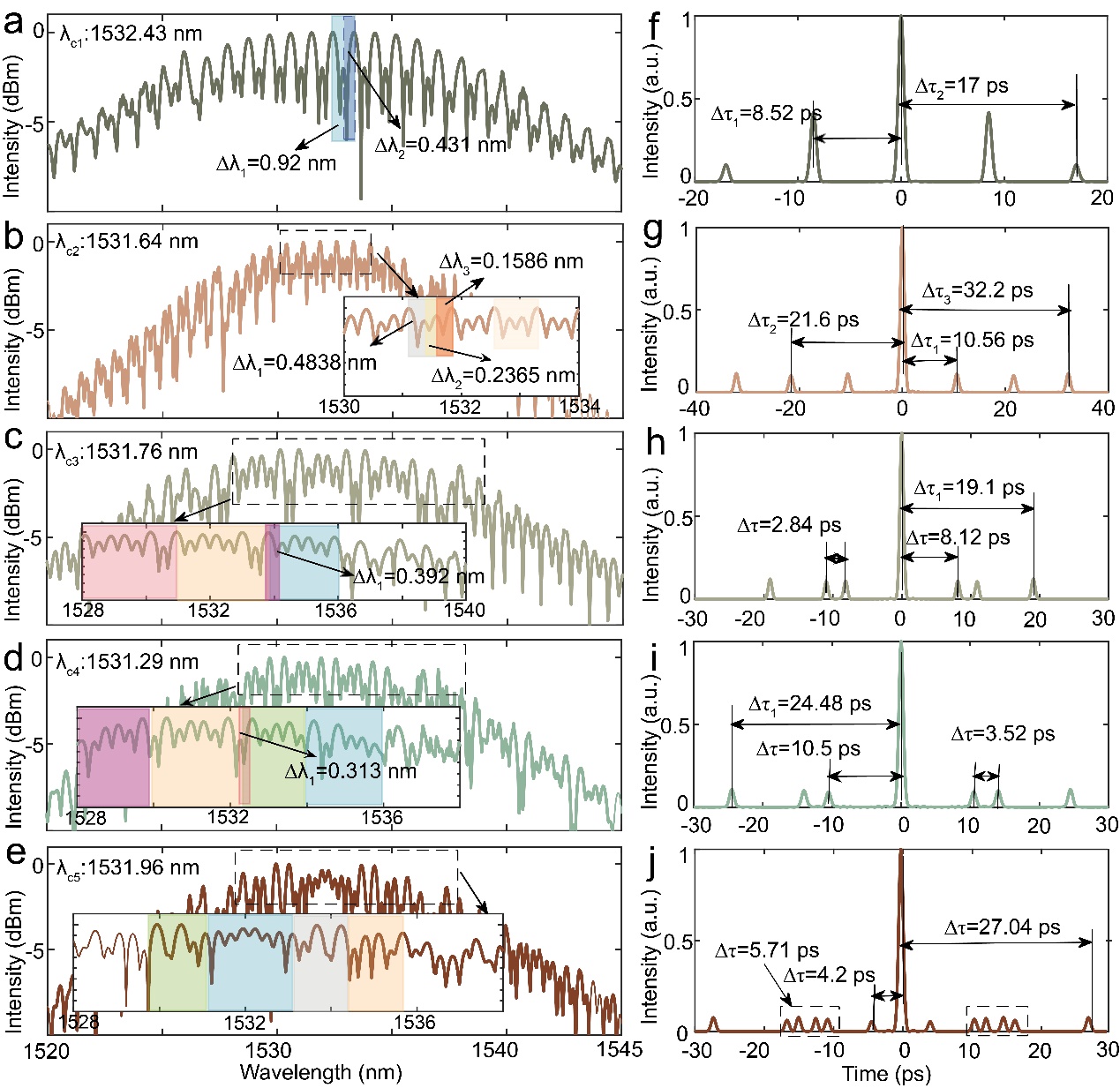


**Fig. S2** **a-j** Simulation spectra of PHEOD bound multi-solitons with different conditions. **f-j** Corresponding autocorrelation traces.

The formation of bound solitons can be attributed to the balance of attraction and repulsion between solitons introduced by the soliton-continuum interaction, which is a periodical function with a series of equilibrium points^7^. Our experimental results show that the identical phenomenon can be realized through the intra-cavity high-order dispersion management and the adjustment of the PC while maintaining a constant pump power. Figure S2a-j depicts the spectra of PHEOD bound tri-soliton, four-soliton, and seven-soliton, along with their corresponding autocorrelation traces under different values of intra-cavity *β_8_*. Unlike PHEOD bound soliton pairs, the spectral modulation of PHEOD bound multi-soliton is no longer singular. A distinct secondary modulation (light blue rectangle) can be observed in Fig. S2a, with modulation periods of 0.92 nm (light blue rectangle) and 0.431 nm (dark blue rectangle). The corresponding pulse separations in Fig. S2f are 8.52 ps and 17 ps. Furthermore, the spectra in Fig. S2b exhibit a distinct cubic modulation, with modulation periods of 0.4838 nm (gray rectangle), 0.2365 nm (yellow rectangle), and 0.1586 nm (orange rectangle). These correspond to pulse separations of 10.56 ps, 21.6 ps, and 32.2 ps in Fig. S2g. It is noteworthy that the intensity difference of PHEOD solitons within PHEOD bound multi-solitons results in an intensity ratio of autocorrelation traces close to 1:3.5:8.3:3.5:1 and 1:0.9:0.9:8:0.9:0.9:1. These unusual structural bound multi-solitons can be achieved by tuning the PC and intra-cavity high-order dispersion without increasing the pump power.

Similarly, adjusting the intra-cavity β_8_ and loss can realize different intervals between solitons, that is, PHEOD bound four-solitons with different combination states, as present in Fig. S2hi. Figure S2cd gives the corresponding spectra, and it can be seen that the spectrum has two cubic modulations (color rectangle), which is similar to the results shown in Fig. S2b, but more complex. The series of modulation peaks between adjacent maximum peaks shown in Fig. S2e illustrates the complex interaction between the PHEOD soliton within the bound state. The corresponding autocorrelation traces in Fig. S2j consist of four units, two of which are double PHEOD soliton bound states and the other two are single PHEOD soliton. The intensity ratio of different peaks is close to 1:0.8:1:0.9:0.9:0.7:12:0.7:0.9:0.9:1:0.8:1, suggesting that the unusual structural PHEOD bound multi-soliton may be unstable, with variable pulse intensity and separation^9^. Such unusual structural PHEOD bound multi-solitons further substantiate the possibility of changing the number of PHEOD solitons through high-order dispersion management without adjusting the pump power.

**Supplementary References**

1. Tang, D. Y. et al. Soliton interaction in a fiber ring laser. *Physical Review E* **72**, 016616 (2005).

2. Malomed, B. A. Bound solitons in the nonlinear Schrodinger-Ginzburg-Landau equation. *Physical Review A* **44**, 6954-6957 (1991).

3. Malomed, B. A. Bound states of envelope solitons. *Physical review E* **47**, 2874-2880 (1993).

4. Gui, L. L. et al. Observation of various bound solitons in a carbon-nanotube-based erbium fiber laser. *Journal Of The Optical Society Of America B-Optical Physics* **30**, 158-164 (2013).

5. Komarov, A. et al. Quantization of binding energy of structural solitons in passive mode-locked fiber lasers. *Physical Review A* **79**, 033807 (2009).

6. Chen, Z. et al. Switchable and Reciprocal Soliton Bound States Enabled by Continuously Tunable Local Modal-Birefringence in a Mode-Locked Fiber Laser. *IEEE Journal Of Quantum Electronics* **59**, 1-7 (2023).

7. Zhu, T., et al. Observation of controllable tightly and loosely bound solitons with an all-fiber saturable absorber. *Photonics Research* **7**, 61-68 (2018).

8. Zhang, X. et al. Spatiotemporal self-mode-locked operation in a compact partial multimode Er-doped fiber laser. *Optics Letters* **47**, 2081-2084 (2022).

9. Zhang, D., et al. SnS_2_ Microsheets for Optical Supramolecular Generation. *Annalen der Physik* **534**, 2200055 (2022).
